# A Variable Step-Size FxLMS Algorithm for Nonlinear Feedforward Active Noise Control

**DOI:** 10.3390/s25082569

**Published:** 2025-04-18

**Authors:** Thi Trung Tin Nguyen, Faxiang Zhang, Jing Na, Le Thai Nguyen, Gengen Li, Altyib Abdallah Mahmoud Ahmed

**Affiliations:** 1 Yunnan Key Laboratory of Intelligent Control and Application, Faculty of Mechanical & Electrical Engineering, Kunming University of Science & Technology, Kunming 650500, China; 20201103008@stu.kust.edu.cn (T.T.T.N.); najing25@kust.edu.cn (J.N.); 20223103005@stu.kust.edu.cn (G.L.); 20201103007@stu.kust.edu.cn (A.A.M.A.); 2 Faculty of Engineering and Technology, Nguyen Tat Thanh University, Ho Chi Minh City 700000, Vietnam; nlthai@ntt.edu.vn

**Keywords:** active noise control, filtered-x least-mean-square algorithm, variable step-size learning, adaptive neuro-fuzzy network, nonlinear path

## Abstract

Active noise control (ANC) represents an efficient technology for enhancing the noise suppression performance and ensuring the stable operation of multi-sensor systems through generative model-enhanced data representation and dynamic information fusion across heterogeneous sensors due to the complexity of the real-world environment. To address problems caused by a nonlinear noise source, a novel adaptive neuro-fuzzy network controller is proposed for feedforward nonlinear ANC systems based on a variable step-size filtered-x least-mean-square (VSS-LMS) algorithm. Specifically, the LMS algorithm is first introduced to update the weight parameters of the controller based on the adaptive neuro-fuzzy network. Then, a variable step-size adjustment strategy is proposed to calculate the learning gain used in the LMS algorithm, which aims to improve the nonlinear noise suppression performance. Additionally, the stability of the proposed method is proven by the discrete Lyapunov theorem. Extensive simulation experiments show that the proposed method surpasses the mainstream ANC methods with regard to nonlinear noise.

## 1. Introduction

The increasing demand for automated perception and operation, driven by generative model-powered multimodal data synthesis and adaptive information fusion frameworks, has spurred studies and applications in multi-sensor systems [1,2]. However, various noises may disrupt the stable operation of multi-sensor systems. To tackle this problem, an efficient noise control algorithm [3,4], which can eliminate the noise interference of original signals, is required in various real-world scenarios. This has inspired a great deal of research on noise control over the past decades.

In general, there are two noise control methods: passive noise control (PNC) and active noise control (ANC). The PNC technique is based on the physical properties of materials capable of absorbing sound to reduce sound propagation. Owing to its advantages, this method has been successfully applied to a great deal of civil, industrial, and military equipment. However, it is challenging to deploy these technologies in specific scenarios due to low-frequency sound, dynamic changes, and limited space [5,6,7].

To overcome the limitations of the PNC method, the ANC system utilizes a secondary anti-noise signal, which has the same amplitude and opposite phase as the original noise, to cancel out the noise by employing the principle of signal superposition. This approach has been recognized as an effective method for reducing low-frequency noise and has shown significant potential in recent decades due to its flexibility and ease of integration on various platforms [8,9]. For this reason, researchers have explored numerous ANC strategies over the past decades [10,11]. According to the differences in the basic model structures, the current ANC can be roughly classified into feedforward ANC (FF-ANC) algorithms [12,13] and feedback ANC algorithms [14,15]. FF-ANC algorithms rely on a reference sensor to obtain the noise source. In contrast, feedback ANC algorithms only use the error signal to eliminate the noise signal in order to address the limitations of FF-ANC algorithms [16,17]. In recent ANC systems, the noise sources can be predicted by using an acoustic sensor. Therefore, the FF-ANC has led to a new trend in the ANC problem due to its powerful performance. Representative methods include the Wiener filter [12], the compensator structure that combines the infinite impulse response (IIR) filter and the finite impulse response (FIR) filter [18], the filtered-x least-mean-square (FxLMS) algorithm [19,20], the filtered-x normalized least mean square (FxNLMS) algorithm [21], and the filtered-s least mean square (FsLMS) algorithm [22]. Furthermore, more complex techniques have also been proposed for ANC systems, such as the approach in [23], which presented an adaptive bilinear filter to replace the adaptive FIR filter for nonlinear FF ANC systems. Although the control performance is improved, the computational costs are too high when the order of the filter increases. Then, adaptive recursive second-order Volterra (RSOV) filters have been introduced [24] to solve the problems of signal saturation and nonlinearities in the ANC systems. Again, although the performance is highly reliable, the computational complexity also increases significantly. Moreover, multilayer perceptron networks [25] were also used to handle the nonlinearities in the noise transmission paths, but this proposed method showed that the convergence speed is slow and the calculation load is increased. Additionally, multichannel methods have also been introduced for nonlinear ANC systems [26,27,28]. Scott C. Douglas [26] introduced a multi-channel control model by using the FxLMS algorithm applied to a broadband ANC system, and the obtained results were reliable. The authors [27] utilized a random Fourier filter to replace the adaptive FIR filter-based nonlinear multi-channel narrowband ANC system, and the simulation results indicate that the performance of the random Fourier filter method based on the FxLMS algorithm is superior to that of the conventional FxLMS algorithm. The incremental-learning-based adaptive filter [28] was applied to multichannel nonlinear ANC systems, which also attains higher efficiency than conventional ANC systems. Generally, multi-channel ANC systems provide reliable performance, but the computational load increases significantly as the number of channels rises. On the other hand, the FxLMS algorithm has been applied to the ANC systems [26,29,30,31] because of its simplicity in the designed system. To improve the convergence rate, a variable step size based on the squared error has been introduced to estimate the unknown transfer function [32,33], where the simulation results show that the convergence speed is significantly improved compared with the classical FxLMS algorithm. Following this idea, some authors have developed the variable step-size FxLMS algorithm for ANC systems [34,35,36,37,38,39]. However, FF-ANC methods only focus on improving the structure of the ANC system with the FxLMS algorithm (which utilizes an FIR filter structure and the LMS learning algorithm to optimize the weights of the FIR filter), and it is performed on noise sources and the primary path with minor nonlinearity or even with linear dynamics. Therefore, for practical ANC systems with FxLMS algorithms, the nonlinearities in the primary path, noise sources, and even the secondary path should be further addressed to enhance the control performance [22].

To solve the above challenge, an adaptive neuro-fuzzy network (ANFN) based on the FxLMS algorithm is proposed for the nonlinear FF-ANC systems. Neural networks and fuzzy logic are widely used in nonlinear control, especially in system identification and modeling, control of highly complex systems, and signal processing. While neural networks [40,41] learn from data but operate as a “black box”, fuzzy logic [42,43] uses rule-based reasoning to mimic human expertise. In contrast with fuzzy logic, which lacks self-learning capabilities, neural networks can adapt dynamically to changing environments. Additionally, with the rapid advancement of technology today, DSPs with high processing speeds and low costs are being developed, making the testing of complex systems easier. The validation of modern control methods has been conducted on real-time systems, such as Fuzzy Logic Control for hydroponic systems in an Internet of Things (IoT) environment [44] and for the control of switched reluctance motors [45]. Furthermore, recently, neural networks have also been used to update the weights of the FxLMS algorithm, which has been implemented on real ANC systems [46,47]. To account for uncertainties and nonlinearities in ANC systems, an adaptive neural network approach is proposed. At the same time, a fuzzy logic-based strategy is introduced to overcome the limitations associated with manual fine-tuning and the nonlinear behavior of conventional methods. The integration of these two approaches exploits their respective advantages and enables a more efficient convergence to optimal solutions. The proposed ANFN method can handle the case where noise sources and primary paths have high nonlinearities. Then, we develop a novel controller based on the VSS-LMS algorithm [32], which has a lower computational load and adaptively adjusts the step-size constant used as the learning gain. The convergence of these ANC systems is also proven using the Lyapunov function. Finally, extensive comparative simulations are performed to validate the proposed methods. However, for nonlinear systems, ensuring their reliability and performance requires a significant amount of computation and is also dependent on the signal’s sampling frequency. Therefore, to experiment with nonlinear ANC systems, it is essential to calculate the system’s computational load to select the appropriate DSP sets.

The rest of this paper is organized as follows: Section 2 outlines the basic principle of the FF-ANC system and presents the detailed design of nonlinear ANC systems using the ANFN controller. In Section 2.4, suitable comparisons with the available results are examined to address the computational complexities. Simulations are shown in Section 3. Section 4 draws some conclusions.

## 2. Adaptive Neuro-Fuzzy Network ANC System

### 2.1. Traditional ANC System

**Figure 1:**
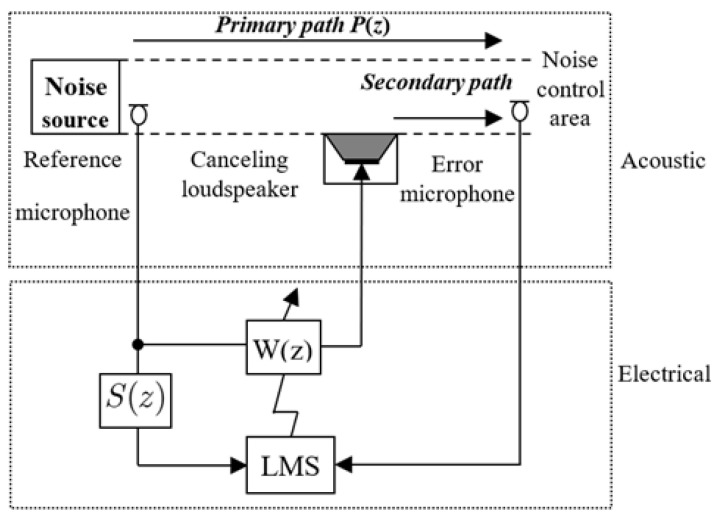
Schematic of ANC systems.

**Figure 2:**
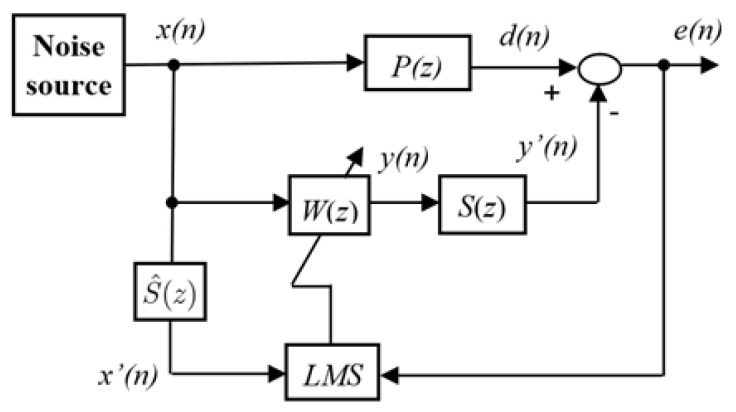
Block diagram of ANC with the FxLMS algorithm.

As shown in Figure 1, the principle of ANC systems involves a loudspeaker operated by an adaptive controller, which is combined with the reference signal to cancel the undesired noise. For the traditional ANC system based on the FxLMS algorithm as given in Figure 2, P(z) represents the primary path from the noise source x(n) to the noise control area d(n), while S(z) is the secondary path from the output y(n) of the adaptive controller W(z) to the secondary noise $y^{\prime }\left(n\right)$. Note that S(z) can be obtained by the off-line identification; i.e., we can denote $S\left(z\right)=\hat{S}\left(z\right)=s_{0}+s_{1}z^{-1}+\ldots +s_{J}z^{-J}$. Moreover, we define the residual noise as

(1) $$\begin{array}{c} e\left(n\right)=d\left(n\right)-y^{\prime }\left(n\right). \end{array}$$

where $y^{\prime }\left(n\right)=\sum_{i=0}^{J}s_{j}y\left(n-j\right)$.

The aim of ANC systems is to generate adaptive controller-based secondary noise y(n) to reduce the residual noise e(n) in the noise control area. As shown in Figure 2, the output of the adaptive controller can be expressed as

(2) $$\begin{array}{c} y\left(n\right)=\sum_{i=0}^{N-1}w_{i}\left(n\right)x\left(n-k\right)=w^{T}\left(n\right)X\left(n\right). \end{array}$$

where $w\left(n\right)={\left[\begin{array}{c} w_{0}\left(n\right),w_{1}\left(n\right),\ldots ,w_{N-1}\left(n\right) \end{array}\right]}^{T}$, $X\left(n\right)={\left[x\left(n\right),x\left(n-1\right),\ldots ,x\left(n-N-1\right)\right]}^{T}$, *N* is the order of the adaptive controller, X(n) is the input vector of the adaptive controller, and w(n) are the coefficients of the adaptive filter W(z) that is updated online based on the reference signal and residual noise.

In classical ANC systems, the LMS algorithm is often used to detect the coefficients w(n). For the convenience of system analysis, define $\xi \left(n\right)=E\left[e^{2}\left(n\right)\right]$ as a mean square cost function. Then the coefficients w(n) are updated to minimize the error based on the following update law:

(3) $$\begin{array}{c} w\left(n+1\right)=\;w\left(n\right)-\frac{\mu }{2}\;\nabla \xi \left(n\right), \end{array}$$

where μ>0 represents the learning gain of the adaptive algorithm, and ∇ξ denotes the gradient of the cost function, which is given by

(4) $$\begin{array}{c} \nabla \xi ={\left[\frac{\partial \xi }{\partial w_{0}\left(n\right)},\frac{\partial \xi }{\partial w_{1}\left(n\right)},\ldots ,\frac{\partial \xi }{\partial w_{N-1}\left(n\right)}\right]}^{T}. \end{array}$$

The *k*th component of $▽e^{2}\left(n\right)$ can be derived as follows:

(5) $$\begin{array}{c} \frac{\partial \xi }{\partial w(n)}=2e\left(n\right)\frac{\partial e(n)}{\partial w(n)}. \end{array}$$

Due to the fact that d(n) operates independently of w(n), by substituting $e\left(n\right)=d\left(n\right)-y^{\prime }\left(n\right)$ into (5), it can be rewritten as

(6) $$\begin{array}{c} \frac{\partial \xi }{\partial w(n)}=-2e\left(n\right)\frac{\partial y^{\prime }\left(n\right)}{\partial w(n)}. \end{array}$$

From (2), it can be concluded that

(7) $$\begin{array}{c} \begin{array}{cc} \frac{\partial \xi (n)}{\partial w(n)} & =-2e\left(n\right)\frac{\partial \sum_{j=0}^{J}s_{j}y\left(n-j\right)}{\partial w(n)}\\ & =-2e\left(n\right)\frac{\sum_{j=0}^{J}s_{j}w\left(n\right)X\left(n-j\right)}{\partial w(n)}\\ & =-2e\left(n\right)\sum_{j=0}^{J}s_{j}X\left(n-j\right). \end{array} \end{array}$$

Substituting (7) into (3), the update law can be further shown as

(8) $$\begin{array}{c} w\left(n+1\right)=w\left(n\right)+\mu e\left(n\right)\sum_{j=0}^{J}s_{j}X\left(n-j\right). \end{array}$$

Note that the adaptive law (8) is based on the gradient descent technology; its stability analysis of (8) is shown in Theorem 1.

**Theorem** **1.** *For the ANC system in Figure 2 with the adaptive law* (8), *if the learning gain μ satisfies $0<\mu <2/{∥\sum_{j=0}^{J}s_{j}X\left(n-j\right)∥}^{2}$, then the ANC system is stable.*

**Proof.**  The proof of Theorem 1 can be found in Appendix A. □

Although the above-mentioned traditional ANC system with the FxLMS algorithm can eliminate the noise in the noise control area through the designed control to operate the loudspeaker, it is assumed that the primary path P(z) and the secondary path S(z) are all linear, which may not be true in practice. Furthermore, the learning gain μ>0 used in the adaptive algorithm, although it meets the condition given in (A5), is set as a constant, which may in turn limit the control performance of these traditional ANC systems.

### 2.2. Nonlinear ANC System with the FxLMS Algorithm

**Figure 3:**
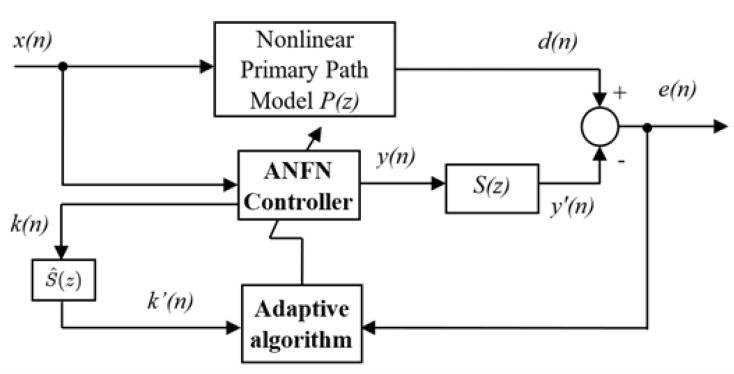
Nonlinear ANFN ANC system.

**Figure 4:**
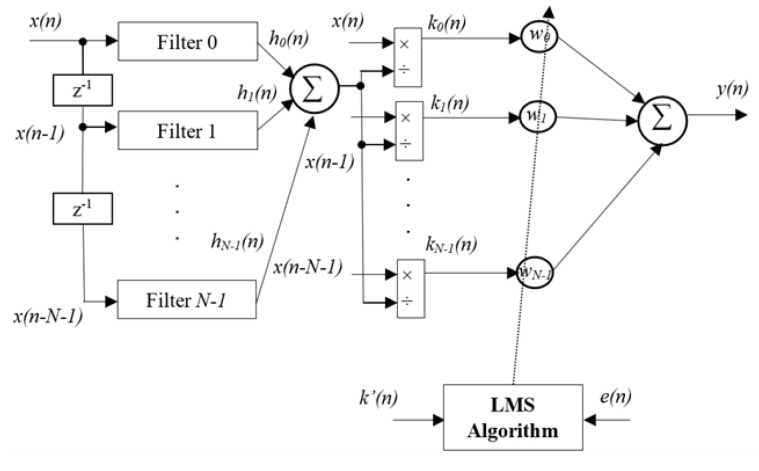
Diagram of adaptive nonlinear ANFN-controller using the FxLMS algorithm.

In this subsection, an alternative nonlinear ANC method will be presented for addressing the nonlinear dynamics in the traditional ANC system based on the FxLMS algorithm, whose block diagram is shown in Figure 3. It is shown that an ANFN controller is utilized to address the nonlinearities in the primary path model, thereby enhancing the control response. The detailed controller can be found in Figure 4, in which an NN is adopted as the control to handle the induced nonlinearities.

As shown in Figure 4, the proposed ANFN model is constructed with five layers, while denoting $O_{i}^{\left(q\right)}$ as the output for the *i*th component of the layer *q*.

Layer 1: Layer 1, known as the input layer, is the place where nodes initially receive signals from various channels. These signals are then directly passed on to Layer 2, In this class, we use the i-function delay (where *i* = 0 to *N*− 1) to split the serial input signal sequence into parallel signal sequences, which can be represented as follows:

(9) $$\begin{array}{c} O_{i}^{\left(1\right)}=x\left(n-i\right). \end{array}$$

Layer 2: In this layer, a membership function employs Gaussian distribution functions as a signal filter for handling nonlinear components, as illustrated below:

(10) $$\begin{array}{c} O_{i}^{\left(2\right)}=h_{i}\left(n\right)=O_{i}^{\left(1\right)}\cdot ae^{\frac{-{\left(O_{i}^{\left(1\right)}-c\right)}^{2}}{2\sigma^{2}}}, \end{array}$$

where *a* represents the amplitude (or height) of the Gaussian curve, which scales the overall function; *c* represents the mean (or center) of the distribution and determines the location of the peak of the Gaussian; and σ represents the standard deviation, which controls the width of the Gaussian curve (a smaller σ makes the curve narrower, while a larger σ makes it wider). These parameters are optimized by means of basic algorithms such as steepest descent, as well as more advanced algorithms like least-squares recursion and Levenberg–Marquardt, so as to enhance the learning process. However, in order to reduce the computational process while still fulfilling the requirements of the ANC system, this paper pre-estimates the parameters of the Gaussian function, and the architecture remains fixed without any adjustments during system operation.

Layer 3: In this layer, the summation operation carried out in Layer 2 is calculated as follows:

(11) $$\begin{array}{c} O^{\left(3\right)}=\sum_{i=0}^{N-1}O_{i}^{\left(2\right)}. \end{array}$$

Layer 4: Each node in this layer is responsible for normalizing the output of a node in the previous layer.

(12) $$\begin{array}{c} O_{i}^{\left(4\right)}=k_{i}\left(n\right)=\frac{x(n-i)}{O^{\left(3\right)}}. \end{array}$$

Layer 5: Layer 5 is designated as the output layer. The output of this layer combines all components from Layer 4 and uses the LMS algorithm to update the coefficients of w(n), as follows:

(13) $$\begin{array}{c} \begin{array}{cc} O^{\left(5\right)} & =y(n)\\ & =\sum_{i=0}^{N-1}w_{i}\left(n\right)k_{i}\left(n\right)\\ & =w^{T}\left(n\right)k\left(n\right)\\ \; & =\frac{1}{\sum_{i=0}^{N-1}x\left(n-i\right)\cdot ae^{\frac{-0.5{\left(x(n)-c\right)}^{2}}{\sigma^{2}}}}w^{T}\left(n\right)X\left(n\right). \end{array} \end{array}$$

where $w^{T}\left(n\right)$ is the transpose vector of w(n), which can be updated online to optimize the residual noise $\xi \left(n\right)=e^{2}\left(n\right)$.

Similar to (3), the adaptive law can be designed as follows:

(14) $$\begin{array}{c} w\left(n+1\right)=w\left(n\right)-\frac{\mu }{2}\nabla \xi \left(n\right), \end{array}$$

where it follows that

(15) $$\begin{array}{c} \frac{\partial \xi (n)}{\partial w(n)}=\frac{\partial \xi (n)}{\partial e(n)}\cdot \frac{\partial e(n)}{\partial y^{\prime }\left(n\right)}\cdot \sum_{j=0}^{J}\left(\frac{\partial y^{\prime }\left(n\right)}{\partial y(n-j)}\cdot \frac{\partial y(n-j)}{\partial w(n)}\right), \end{array}$$

(16) $$\begin{array}{c} \frac{\partial \xi (n)}{\partial c(n)}=2\varepsilon \left(n\right),\;\frac{\partial e(n)}{\partial y^{\prime }\left(n\right)}=-1,\;\frac{\partial y^{\prime }\left(n\right)}{\partial y(n-j)}=s_{j},\;\frac{\partial y(n-j)}{\partial w(n)}=k\left(n-j\right), \end{array}$$

Based on (15) and (16), one has that

(17) $$\begin{array}{c} \frac{\partial \xi (n)}{\partial w(n)}=-2\epsilon \left(n\right)\sum_{j=0}^{J}s_{j}k\left(n-j\right), \end{array}$$

Finally, we can obtain the following adaptive law:

(18) $$\begin{array}{c} w\left(n+1\right)=w\left(n\right)+\mu e\left(n\right)\sum_{j=0}^{J}s_{j}k\left(n-j\right). \end{array}$$

**Theorem** **2.** *For the ANC system in Figure 3 with the adaptive law* (18), *if the learning gain μ satisfies $0<\mu <2/{∥\frac{\sum_{j=0}^{J}s_{j}X\left(n-j\right)}{\sum_{i=0}^{N-1}x\left(n-i\right)\cdot ac^{\frac{-0.5{\left(x\left(n\right)-c\right)}^{2}}{\sigma^{2}}}}∥}^{2}$, then the ANC system is stable.*

**Proof.**  Similar to the proof for Theorem 1, based on the Lyapunov function $V\left(n\right)=\frac{1}{2}e^{2}\left(n\right)=\frac{1}{2}{\left(d\left(n\right)-y^{\prime }\left(n\right)\right)}^{2}$ in (A1) and the result obtained in (17), it can be concluded that

(19) $$\begin{array}{c} \Delta V\left(n\right)=-\frac{1}{2}\;\mu e^{2}\left(n\right){∥\sum_{j=0}^{J}s_{j}k\left(n-j\right)∥}^{2}\cdot \left(2-\mu {∥\sum_{j=0}^{J}s_{j}k\left(n-j\right)∥}^{2}\right), \end{array}$$

Therefore, one has

(20) $$\begin{array}{c} 0<\mu <2\;/{∥\sum_{j=0}^{J}s_{j}k\left(n-j\right)∥}^{2}, \end{array}$$

where k(n) is the signal extracted from layer 4 of the ANFN controllers. From (9) to (12), we can obtain k(n) as follows:

(21) $$\begin{array}{c} k\left(n\right)=\frac{x(n)}{\sum_{i=0}^{N-1}x\left(n-i\right)\cdot ae^{\frac{-0.5{\left(x\left(n\right)-c\right)}^{2}}{\sigma^{2}}}}. \end{array}$$

Consequently, we obtain the condition to ensure the stability of the ANC system based on the ANFN controller as follows:

(22) $$\begin{array}{c} 0<\mu <2/{∥\frac{\sum_{j=0}^{J}s_{j}X\left(n-j\right)}{\sum_{i=0}^{N-1}x\left(n-i\right)\cdot ae^{\frac{-0.5{\left(x\left(n\right)-c\right)}^{2}}{\sigma^{2}}}}∥}^{2}. \end{array}$$

□

**Remark** **1.** *In practice, the learning gain plays a crucial role. When the noise source cannot be ignored, reducing μ will result in a slower convergence rate. On the other hand, selecting a large coefficient μ may lead to system instability as proved in Theorem 2. To address this issue, a varying step-size method will be presented in the following subsection. Additionally, the convergence conditions of the system will also be provided.*

### 2.3. Nonlinear ANC System with the VSS-LMS Algorithm

To further improve the convergence of the proposed adaptive law (18), the VSS-LMS algorithm is introduced in this subsection. The basic idea of the VSS algorithm is to utilize a variable step-size at the commencement of the noise-reduction process to accelerate noise reduction and gradually diminish the adaptive gain until the system converges, thereby enhancing system performance. This approach not only augments the initial response time but also guarantees the system’s stability during the adaptation process. By dynamically fine-tuning the step-size, the VSS-LMS algorithm effectively balances rapid convergence with sustained performance and simultaneously realizes noise reduction.

The step size μ(n) is updated online as follows:

(23) $$\begin{array}{c} \mu \left(n\right)=\left\{\begin{array}{cc} \mu_{\max}, & \mathrm{if}\;\mu^{\prime }\left(n\right)>\mu_{\max}\\ \mu^{\prime }\left(n\right), & \mathrm{otherwise} \end{array}\right.. \end{array}$$

From Theorem 2, it is known that there is a saturation operator $\mu_{max}$, and the parameter $\mu^{\prime }\left(n\right)$ is necessary for maintaining the stability of the ANC system. The step size $\mu^{\prime }\left(n\right)$ is subsequently defined as

(24) $$\begin{array}{c} \mu^{\prime }\left(n+1\right)=\alpha \mu^{\prime }\left(n\right)+\gamma e^{2}\left(n\right),\;\mu^{\prime }\left(0\right)\geq 0, \end{array}$$

where γ>0 is a weight of the current error, and 0<α<1 is a forgetting factor that ensures that the learning gain $\mu^{\prime }$ is bounded.

It is evident that the fact of $\mu^{\prime }\left(n\right)>0$ can be established due to $\mu^{\prime }\left(n\right)\geq 0$. Based on (24), we can conclude that the step size increases with a larger control error e(n), facilitating a faster convergence of w(n). On the other hand, as the control error e(n) converges to zero, the step size μ can remain constant to prevent misadjustments.

Next, it can be proven that the step size $\mu^{\prime }\left(n\right)$ is bounded for a sufficiently small γ>0, and e(n) converges to zero.

**Theorem** **3.** *For the ANC system in Figure 3 with the adaptive law* (18) *and a variable step-size algorithm* (23), *there always exists a sufficiently small γ such that the ANC system is stable.*

**Proof.**  From Theorems 1 and 2 and (23), the ANC system is stable if we set $\mu_{\max}\leq 2\;/{∥\sum_{j=0}^{J}s_{j}k\left(n-j\right)∥}^{2}$, such that e(n) will converge to zero under this condition. Next, we further prove that the proposed variable step size μ is bounded. Without limiting generality, we assume that the error e(n) of the system is bounded, that is, |e(n)|≤ε, where ε>0 is a small positive constant.

In this case, with any initial condition $\mu^{\prime }\left(0\right)\geq 0$, we have

(25) $$\begin{array}{c} \begin{array}{cc} \mu^{\prime }\left(1\right) & =\alpha \mu^{\prime }\left(0\right)+\gamma e^{2}\left(0\right)\\ \mu^{\prime }\left(2\right) & =\alpha \mu^{\prime }\left(1\right)+\gamma e^{2}\left(1\right)=\alpha^{2}\mu^{\prime }\left(0\right)+\alpha \gamma e^{2}\left(0\right)+\gamma e^{2}\left(1\right)n\\ \vdots \\ \mu^{\prime }\left(k+1\right) & =\alpha^{k+1}\mu^{\prime }\left(0\right)+\alpha^{k}\gamma e^{2}\left(0\right)+\alpha^{k-1}\gamma e^{2}\left(1\right)+\cdots \alpha \gamma e^{2}\left(k-1\right)+\gamma e^{2}\left(k\right)\\ \vdots \end{array} \end{array}$$

According to the property of a geometric series, $\lim_{n\to \infty }\sum_{k=0}^{n-1}\alpha^{k}=\frac{1}{1-\alpha }$ and $\lim_{n\to \infty }\alpha^{n}=0$ for 0<α<1, which yields that the condition ∣ϵ(n)∣≤ϵ for n=0,1,…,∞ and the initial condition $\mu^{\prime }\left(0\right)\geq 0$ is finite. From (25), one has

(26) $$\begin{array}{c} \mu^{\prime }\left(\infty \right)=\lim_{n\to \infty }\mu^{\prime }\left(n+1\right)\leq \lim_{x\to \infty }\alpha^{n+1}\mu^{\prime }\left(0\right)+\lim_{x\to \infty }\sum_{n=0}^{\infty }\alpha^{n}\gamma \varepsilon =\frac{\gamma \varepsilon }{1-\alpha }. \end{array}$$

From (26), it can be concluded that the variable step size $\mu^{\prime }$ is bounded and can converge to $\mu^{*}=\frac{\gamma \epsilon }{1-\alpha }$. Furthermore, the parameters in (24) can be selected as 0<α<1 and $\gamma \leq \frac{1\alpha }{\epsilon R(n)}$ to ensure that the condition $0<\mu <\frac{2}{1+R(n)}$ holds for n=0,1,…,∞. The stability condition in Theorems 1 and 2 yields that the ANC system is stable. □

### 2.4. Comparisons

To demonstrate the efficiency of the proposed ANFN-VSS-LMS controller for nonlinear ANC systems, as well as the computational effort required, in this section, the computational load of the proposed schemes is compared with that of the conventional ANC using the FxLMS algorithm.

**(1)**  **Traditional ANC system**

The traditional ANC system depicted in Figure 2 utilizes an adaptive FIR filter W(z) and the LMS algorithm for noise control, where *N* represents the length of the adaptive FIR filter. The implementation of the system can be described in Table 1 as follows:

**(2)**  **ANFN controller with the LMS algorithm**

The ANFN controller based on the LMS algorithm is designed in five layers, and the LMS algorithm is utilized to update the adaptive weights of the controller. The calculation process of the system is carried out in Table 2 as follows:

**(3)**  **ANFN controller with the VSS-LMS algorithm**

The implementation of the nonlinear ANFN-VSS-LMS controller is similar to that of the ANFN-LMS controller, with the addition of extra operations required for calculating the variable step size $\mu^{\prime }$ as shown in Table 3.

Table 4 summarizes the computational load of the methods. The proposed ANFN-VSS-LMS method requires higher computational costs because of the use of both the ANFN-LMS controller and the VSS scheme, which are designed to deal with the nonlinearities in the system and enhance the convergence rate.

## 3. Simulations

The sampling frequency of signals is consistently set as 8 kHz, and the linear secondary path model is selected as a signal amplifier [48], i.e.,

(27) $$\begin{array}{c} S\left(z\right)=z^{-2}+1.5z^{-3}+z^{-4}. \end{array}$$

The fuzzy logic system input is defined with nine fuzzy sets. The width *c* and the means $\delta^{2}$ of the Gaussian membership functions are chosen as

(28) $$\begin{array}{c} \begin{array}{cc} c & =\left[-1,-0.75,-0.5,-0.25,0,0.25,0.5,075,1\right],\\ \delta^{2} & =\left[0.4,0.2,0.2,0.2,0.2,0.2,0.2,0.2,0.4\right]. \end{array} \end{array}$$

**Figure 5:**
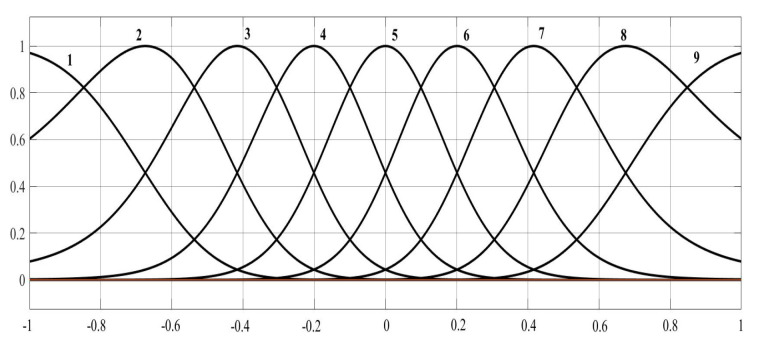
Nine fuzzy sets are Gaussian membership functions of input variables.

These parameters can be chosen through a trial-and-error approach and should be selected to strike a balance between stability and control performance. Figure 5 presents a graphical representation of the means and widths of the Gaussian membership functions. The operating range is assumed to be [0,1].

**Case 1:** In this case, we show that the traditional ANC system using the FxLMS algorithm operates effectively under linear conditions. We select the order of the adaptive filter as N=9, the noise source as a sinusoidal signal with a low frequency of 25 Hz, and the primary path as a linear transmission path [22].

(29) $$\begin{array}{c} d(n)=x(n-3)-0.3x(n-4)+0.2x(n-5). \end{array}$$

Additionally, for the proposed ANFN-LMS, we select μ=0.001 and the ANFN-VSS-LMS method; we select $\mu^{\prime }\left(0\right)=0.0001,\alpha =0.98$ and γ=0.1. The noise is set as x(n)=sin(wn).

**Figure 6:**
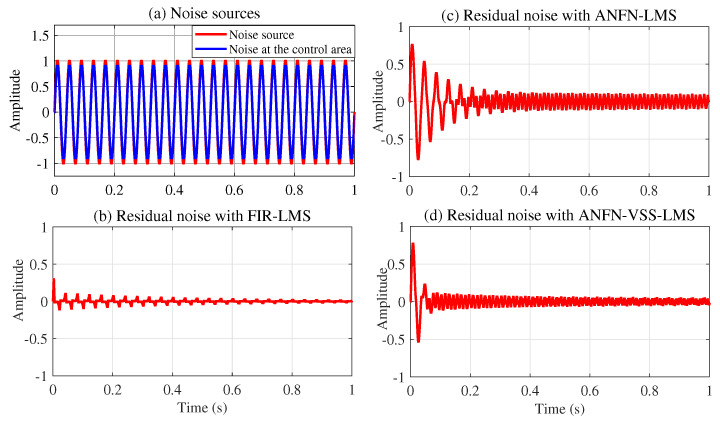
Simulation results for noise with a frequency of 25 Hz in the time domain: (**a**) noise source, (**b**) residual noise of the adaptive linear FIR controller with the LMS algorithm, (**c**) residual noise of the ANFN controller using the LMS algorithm, and (**d**) residual noise of the ANFN controller with the VSS-LMS algorithm.

**Figure 7:**
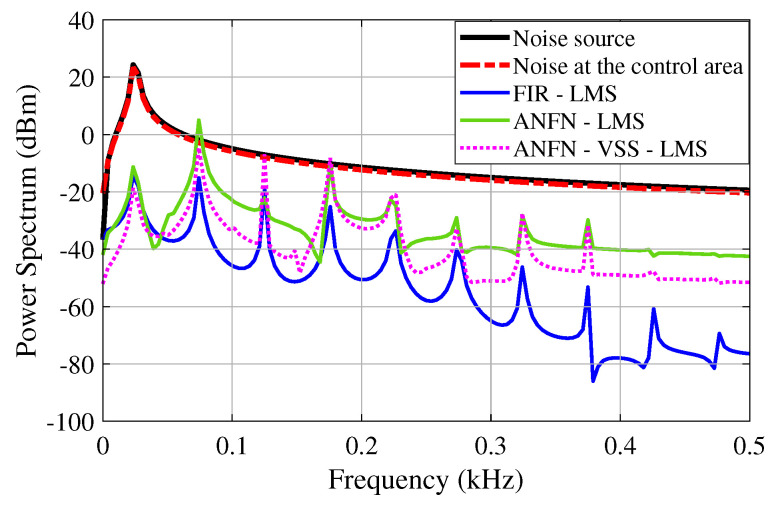
Simulation results for a noise frequency of 25 Hz in the frequency domain.

Figure 6 presents the simulation results in the time domain with a low-frequency noise source x(n) of 25 Hz. Note that Figure 6a shows the noise source to be controlled with the solid red line, and the noise at the control area after being transmitted through the primary path (29) is presented in the solid blue line. This figure indicates that the signal amplitude at the primary path output d(n) is smaller than that of the noise source signal x(n), since all the coefficients in the primary path P(z) are smaller than and equal to one (which is also reasonable as it represents the signal loss in the environment). Figure 6b–d present the residual noise of the three approaches: FIR-LMS, ANFN-LMS, and ANFN-VSS-LMS. Figure 7 shows the simulation results of the three controllers in the frequency domain based on the analysis of 8000 samples. In this figure, the blue solid line displays the power spectrum of the residual noise with the FIR-LMS system, the green solid line shows the power spectrum of the residual noise with the ANFN-LMS system, and the purple dash line represents the power spectrum of the residual noise of the proposed ANFN-VSS-LMS system. From these figures, it can be seen that the performance of the ANFN-VSS-LMS system is better than that of the ANFN-LMS system, and the adaptive FIR-LMS filter is more effective than the proposed ANFN controller in this linear case. This shows that using nonlinear controllers for linear systems is more complex, which also leads to a slower convergence speed compared with linear controllers. However, in reality, we are dealing with nonlinear systems in many cases, as demonstrated by the following simulation cases 2, 3, and 4.

**Figure 8:**
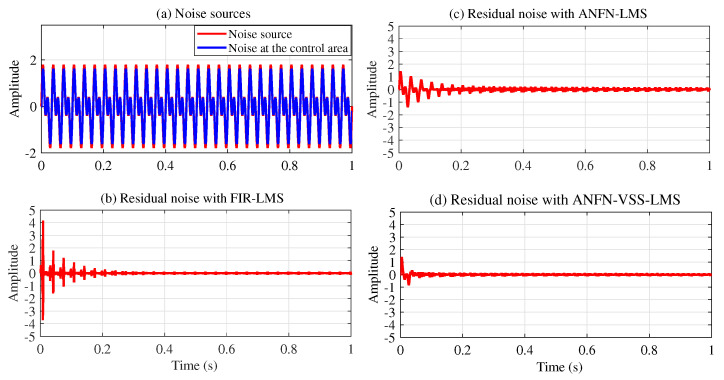
Simulation results for noise with frequencies of 30 Hz and 60 Hz in the time domain: (**a**) noise source, (**b**) residual noise of adaptive FIR controller, (**c**) residual noise of the ANFN controller with the LMS algorithm, and (**d**) residual noise of the ANFN controller with the VSS-LMS algorithm.

**Figure 9:**
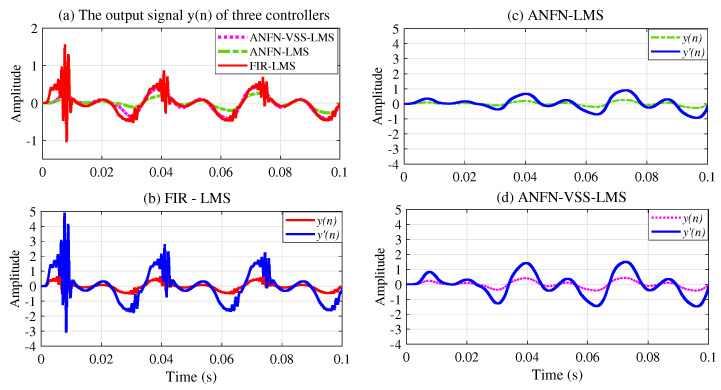
Measurement results of output signals of three controllers for noise frequencies of 30 Hz and 60 Hz in the time domain: (**a**) the output signal y(n) of controllers; (**b**–**d**) the output signal of controllers $y^{\prime }\left(n\right)$ after transmitting the secondary path.

**Figure 10:**
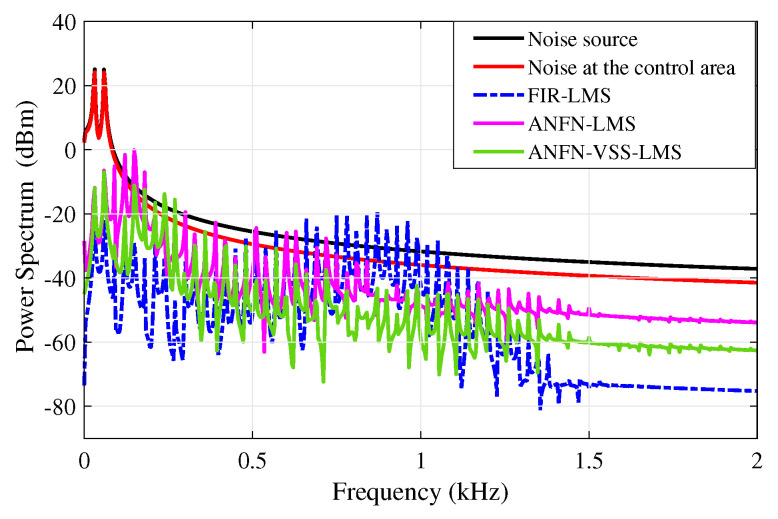
Simulation results for noise frequencies of 30 Hz and 60 Hz in frequency domain.

**Figure 11:**
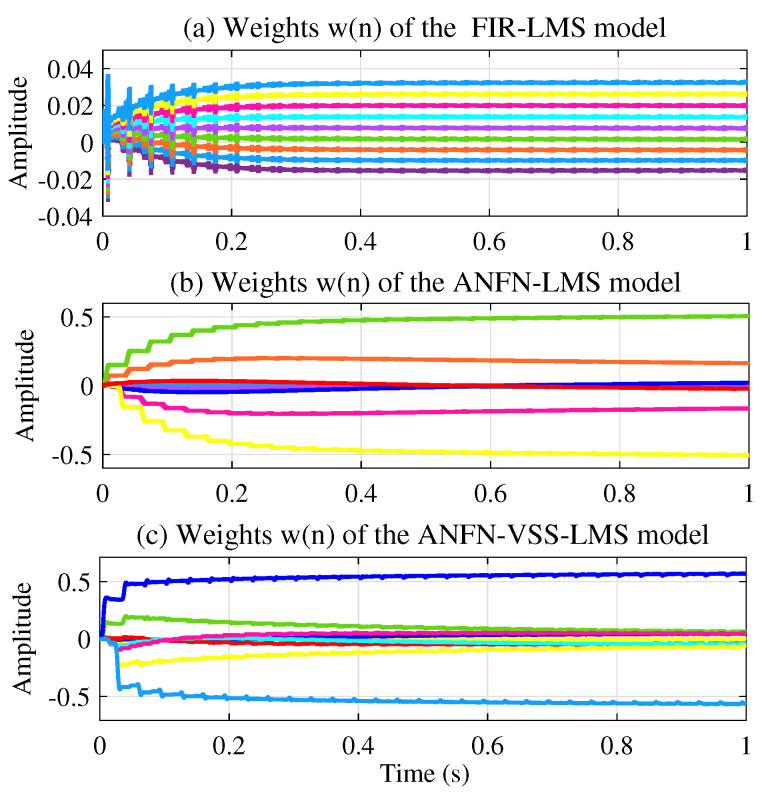
Weights during learning of controllers with frequencies of 30 Hz and 60 Hz.

**Figure 12:**
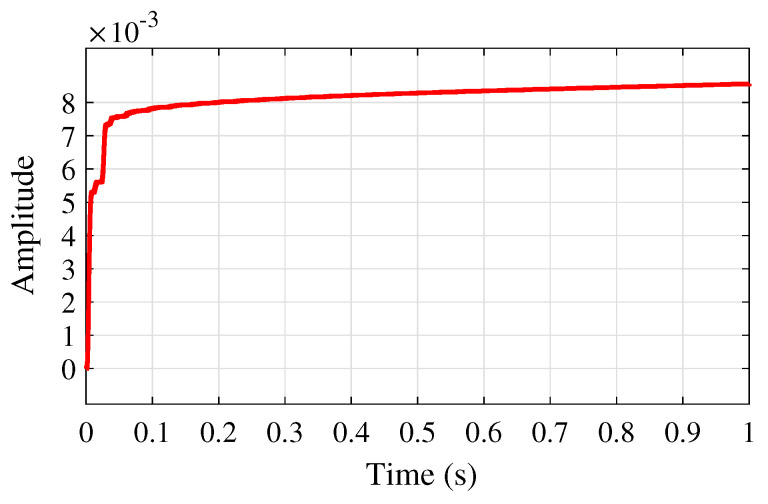
Convergence performance of μ(n).

**Case 2:** In this case, the noise source is selected as a nonlinear signal, which is the sum of two sinusoidal signals with frequencies of 30 Hz and 60 Hz. All other system parameters are identical to those in Case 1. Simulation results in this case are presented in Figure 8, Figure 9, Figure 10, Figure 11 and Figure 12.

Figure 8 depicts the noise source signals and the performance of the ANC system of three methods in the time domain. Figure 8a presents the noise source $x\left(n\right)=\sin\left(\omega_{1}n\right)+\sin\left(\omega_{2}n\right)$ and the noise at the control area d(n). Figure 8b depicts the residual noise of the FIR-LMS filter. Figure 8c and Figure 8d show the residual noise of the ANFN-LMS algorithm and the ANFN-VSS-LMS algorithm, respectively. Here, we can find that the residual noise for FIR- LMS is even larger than a fundamental item. This can be explained as follows: When two signals are added together, in addition to the central frequency components with the largest amplitude, several smaller amplitude signals at other frequencies also appear (called intermodulation products). These products are typically integer multiples or combinations of the original frequencies. Therefore, they can be regarded as nonlinearities of the system, causing ANC systems using FIR-LMS filters to deviate from a stable trajectory or potentially become unstable. To further clarify this issue, we can obtain the output signal y(n) and $y^{\prime }\left(n\right)$ from the secondary path (27), which is shown in Figure 9.

Figure 9a illustrates the output signals y(n) of three controllers, in which the red solid line, the green dash line, and the purple dash line represent the output signals of three controllers: FIR-LMS, ANFN-LMS, and ANFN-VSS-LMS systems, respectively. Here, it can be observed that the ANC system using the FIR-LMS controller generates these undesirable sinusoidal signal components, while the ANFN-LMS and ANFN-VSS-LMS controllers do not show such behavior. Figure 9b–d also present the output signals of the controllers (FIR-LMS, ANFN-LMS, and ANFN-VSS-LMS) $y^{\prime }\left(n\right)$ after the transmission of the secondary path S(z), which indicate that the amplitude of signals $y^{\prime }\left(n\right)$ is further amplified compared with the signals y(n); i.e., the secondary path (27) is selected as a signal amplifier [22]. Therefore, the harmonics [49] in the FIR-LMS controller-based ANC system increase as they pass through the secondary transmission path.

Figure 10 presents the simulation results in the frequency domain, where the black solid line and the red solid line represent the power spectra of the original noise source and the noise in the control area, respectively. The blue dashed line, purple solid line, and green solid line represent the power spectra of the residual noise for the FIR-LMS, ANFN-LMS, and ANFN-VSS-LMS systems, respectively. Here, it can be found that the FIR-based FxLMS method functions well in the frequency range that needs to be controlled, but it also generates signal components outside the frequency range that needs to be controlled (about 0.8 kHz). From this figure, it can be observed that the proposed ANFN-VSS-LMS controller provides a more efficient control response than the ANFN-LMS controller.

Figure 11a presents the learning weights of the FIR-LMS filter, which demonstrates that the system converges. Figure 11b and Figure 11c respectively present the neural network weights of the ANFN-LMS and ANFN-VSS-LMS methods. It can be observed that the learning weights of the ANFN-VSS-LMS algorithm converge more rapidly than those of the ANFN-LMS algorithm. From this figure, it can be seen that the coefficients w(n) of controllers do not converge to fixed values around a fixed point; instead, they remain bounded and show convergent trends. In fact, it can be found from (15) that the coefficients w(n) converge to constant values if and only if e(n)=0.

Figure 12 shows the adaptive learning rate profile of μ(n) based on the proposed VSS-LMS algorithm (23), successfully adjusting the learning rate as needed, which aids in network convergence rather than maintaining a constant value. This figure also illustrates that this step size converges to a constant $\mu_{max}$ as proved in Theorem 3.

To comprehensively evaluate the performance of the proposed method, the quantitative Root Mean Square Error $\left(RMSE=\sqrt{\frac{1}{K}\sum_{i=1}^{K}e_{i}^{2}\left(n\right)}\right)$ is provided in Table 5.

As shown in Table 5, the experimental results indicate that, compared with the ANFN-LMS method, the proposed method reduces the control error index RMSE by 34.61%, and compared with the FIR-LMS method, it reduces by 46.9%. These RMSE values validate that the proposed ANFN-VSS-LMS method can recover the original signal with the smallest residual error MSE, outperforming the established methods.

**Figure 13:**
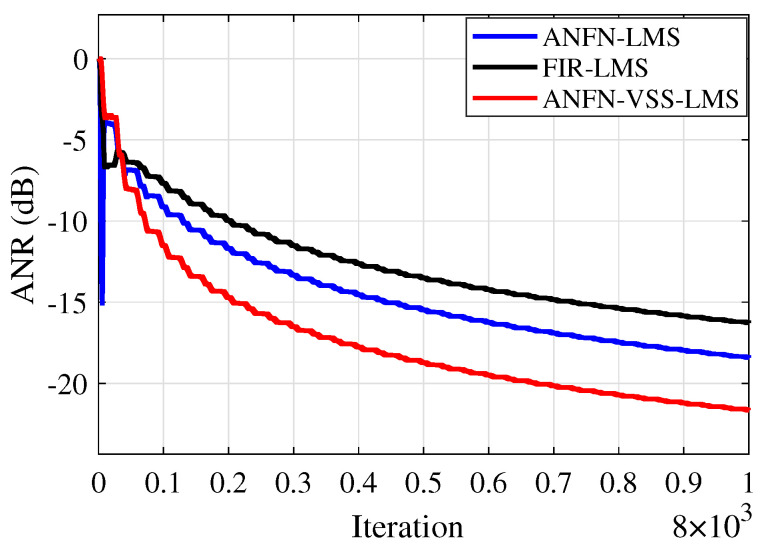
ANR curves of various methods.

Furthermore, to evaluate the effectiveness of the methods, the Average Noise Reduction $\left(ANR\left(dB\right)=10\log_{10}\left\{\frac{E\left[e^{2}\left(n\right)\right]}{E\left[d^{2}\left(n\right)+{y^{\prime }}^{2}\left(n\right)\right]}\right\}\right)$ is also shown in Figure 13. In this case, the proposed ANFN-VSS-LMS system has the lowest steady-state ANR, followed by the ANFN-LMS system and the FIR-LMS model. Among the three models, the proposed ANFN-VSS-LMS system stands out with a significant steady-state advantage compared with the other two models.

**Case 3:** In this case, to show the performance of the proposed ANFN-VSS-LMS method, we use the primary path as a nonlinear transmission line represented by a second-order polynomial as follows:

(30) $$\begin{array}{c} d\left(n\right)=x\left(n-3\right)-0.3x\left(n-4\right)+0.8x^{2}\left(n-4\right)+0.2x\left(n-5\right). \end{array}$$

**Figure 14:**
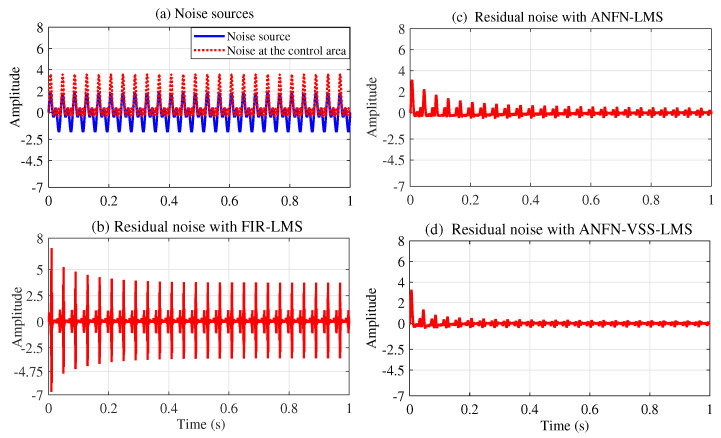
Simulation results for noise with frequencies of 30 Hz and 60 Hz in the time domain.

**Figure 15:**
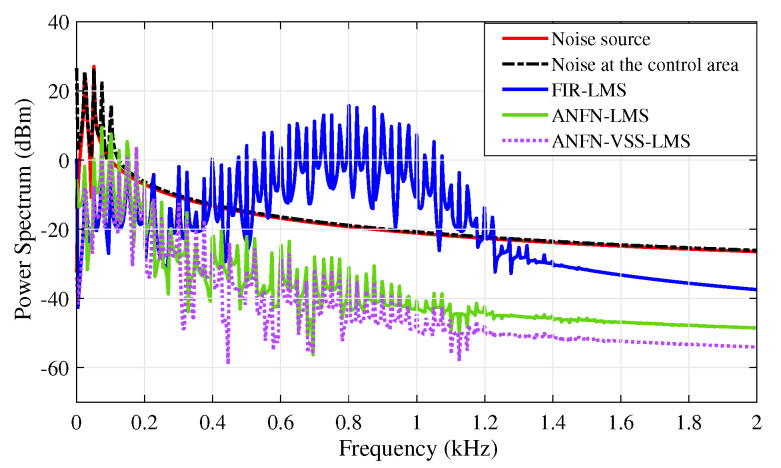
Simulation results for noise frequencies of 30 Hz and 60 Hz in the frequency domain.

**Figure 16:**
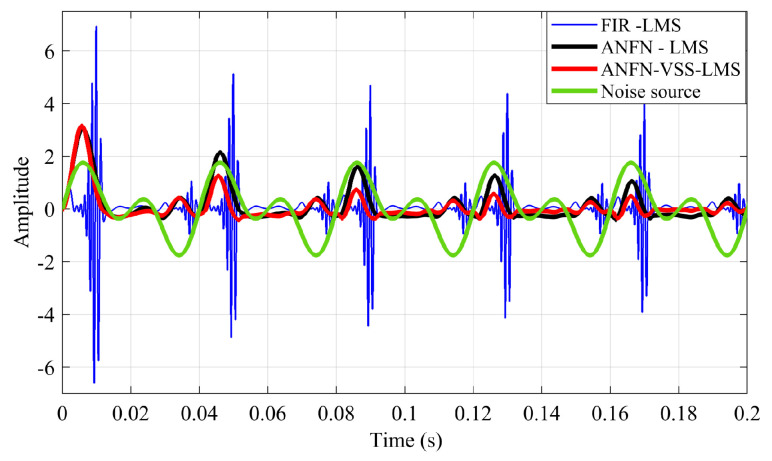
Measurement result of harmonic signals at the output of the controllers.

**Figure 17:**
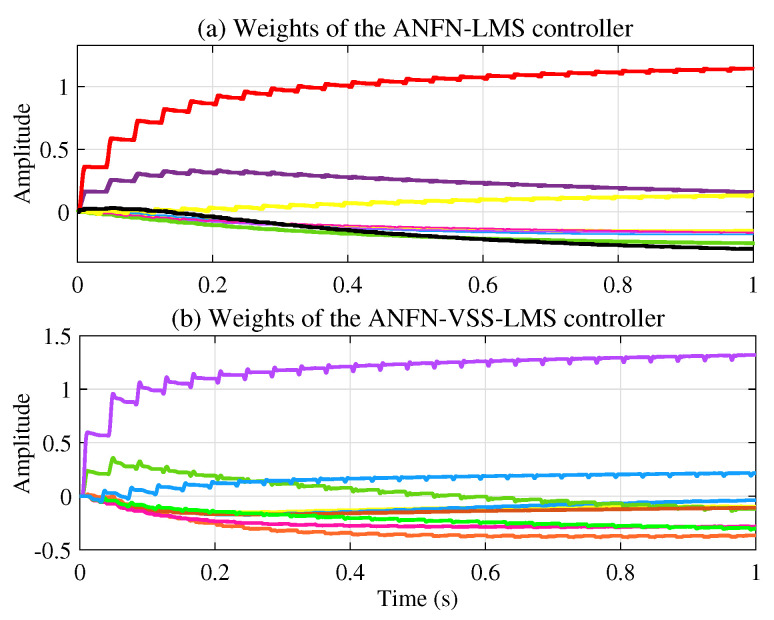
The network weighs during learning of controllers with frequencies of 30 Hz and 60 Hz.

**Figure 18:**
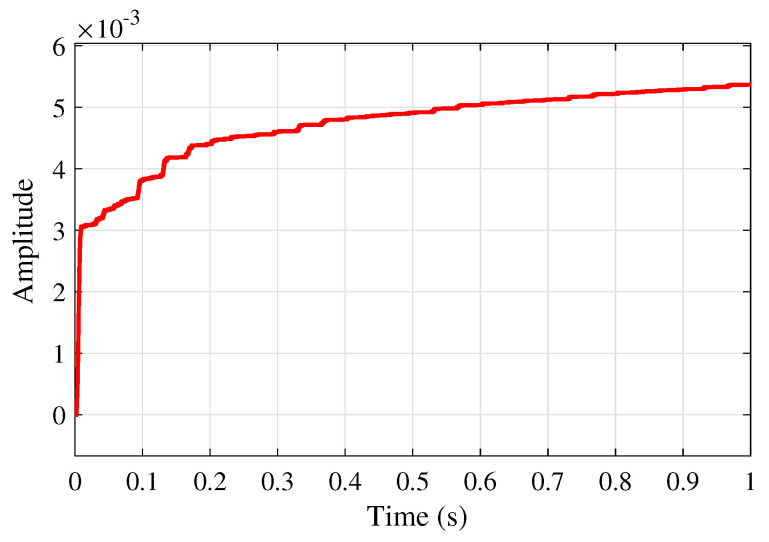
Convergence performance of μ(n).

**Figure 19:**
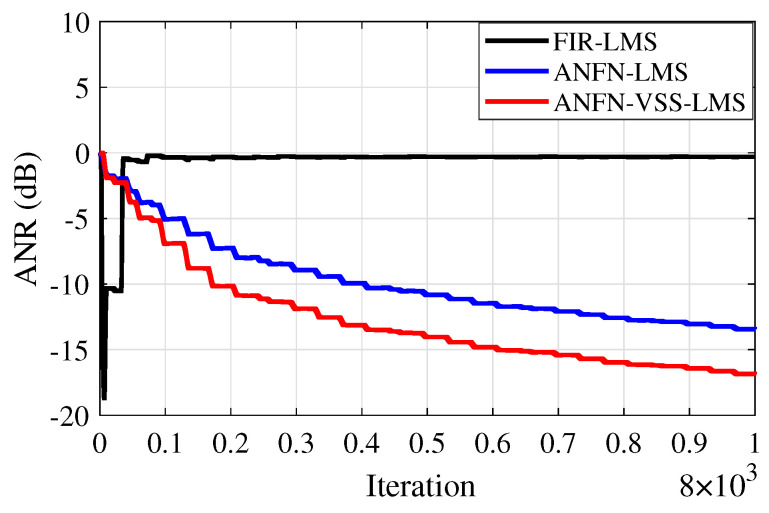
ANR curves of various methods.

All other parameters of the system are chosen to be the same as in Case 2 with the case of $x\left(n\right)=\sin\left(\omega_{n}\right)$. Simulation results are shown in Figure 14, Figure 15, Figure 16, Figure 17, Figure 18 and Figure 19. Figure 14 illustrates the simulation results with a noise source consisting of the sum of two sinusoidal signals with frequencies of 30 Hz and 60 Hz in the time domain. Figure 14a displays the noise source and the noise after transmission through the nonlinear primary path, represented by the solid blue line and the dotted red line, respectively. This figure shows that the amplitude of the signal deformations is quite large compared with the original noise signal. This phenomenon demonstrates that the nonlinear transfer function of the primary path can lead to a nonlinear distortion of the noise [49]. Figure 14b–d depict the residual noise of three approaches: FIR-LMS, ANFN-LMS, and ANFN-VSS-LMS in the time domain. In this figure, the proposed ANFN-VSS-LMS model exhibits the lowest residual noise, followed by the ANFN-LMS model and the model using the adaptive FIR filter. The superior performance of the proposed ANFN-VSS-LMS method stems from its modification of the standard FxLMS algorithm, which dynamically adjusts the step size by incorporating a forgetting factor.

Furthermore, the simulation results in the frequency domain are presented in Figure 15. In this figure, the solid red line and the solid black line respectively represent the power spectrum of the noise source signal to be controlled and the noise signal at the control area after being transmitted through the nonlinear primary path. Here, it is noticed that when the signal travels through a nonlinear transmission line, new frequency components emerge along with the original signal. This effect occurs as the nonlinear properties of the transmission line generate additional frequencies that were not present in the original signal. Additionally, the solid blue line displays the power spectrum of the residual noise from the FIR-LMS model. From this result, it can be seen that the FIR-LMS method fails to converge at a specific frequency of around 0.8 kHz due to the nonlinearities in the transmission path of the ANC system. This emphasizes the ineffectiveness of the adaptive FIR filter for nonlinear ANC systems. The solid green line and the dashed purple line respectively present the power spectrum of the residual noise for the ANFN-LMS controller and the proposed ANFN-VSS-LMS controller. These results indicate that the performance of the proposed ANFN-VSS-LMS controller is more effective than that of the ANFN-LMS controller.

Figure 16 presents the harmonic signals at the output y(n) of three controllers in the time domain. The blue solid line represents the output signal of the FIR-LMS filter, which generates a frequency band different from the original noise frequency. This discrepancy, probably caused by the nonlinearities of the system [49], results in the divergence of the ANC system. The black solid line and the red solid line represent the output signal of the ANFN-LMS controller and the proposed ANFN-VSS-LMS controller, respectively. This indicates that the proposed controllers have successfully removed the harmonic component introduced by the nonlinear noise source and the nonlinear primary path.

Moreover, Figure 17a and Figure 17b show the neural network weights during the learning process of the ANFN-LMS and ANFN-VSS-LMS methods, respectively. It is obvious that the ANFN controller using the VSS-LMS algorithm converges more rapidly than the ANFN controller using the standard LMS algorithm. Figure 18 shows the response of μ(n) for the proposed VSS-LMS method.

Figure 19 illustrates the average noise reduction index of the systems. It is evident from this figure that the proposed ANFN-VSS-LMS method recovers the original signal more effectively than the ANFN-LMS controller. Additionally, the figure shows that the traditional FIR-LMS method fails to recover the original signal.

From the root mean square error indices provided in Table 6, it can be observed that the proposed ANFN-VSS-LMS results in a 30.27% reduction compared with ANFN-LMS and a 96.23% reduction compared with FIR-LMS.

**Figure 20:**
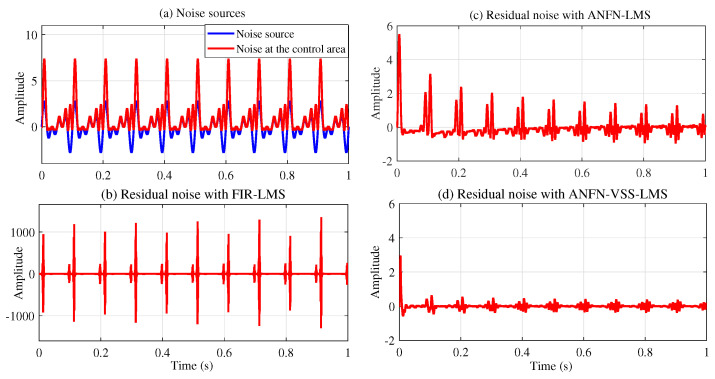
Simulation results for noise frequencies of 20 Hz, 30 Hz, and 40 Hz in time domain.

**Figure 21:**
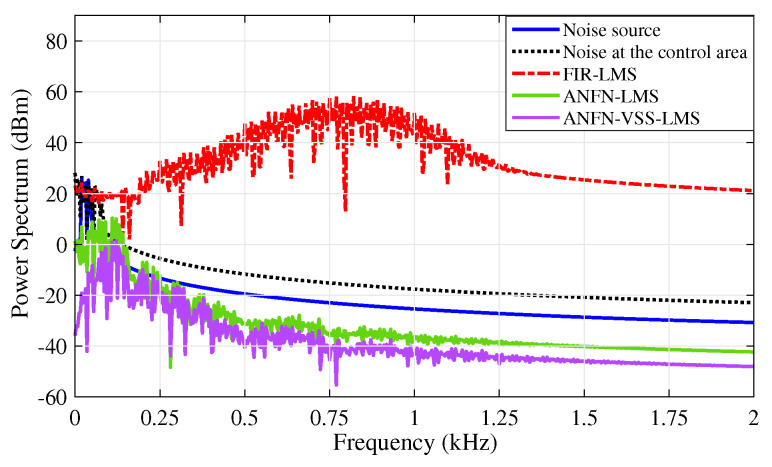
Simulation results for noise frequencies of 20 Hz, 30 Hz, and 40 Hz in the frequency domain.

**Figure 22:**
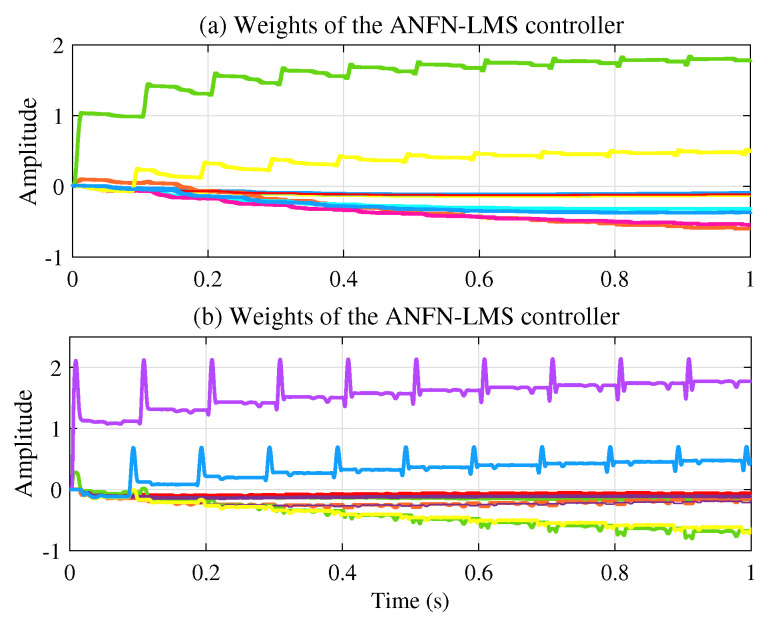
The network weighs during learning of controllers with frequencies of 20 Hz, 30 Hz, and 40 Hz.

**Figure 23:**
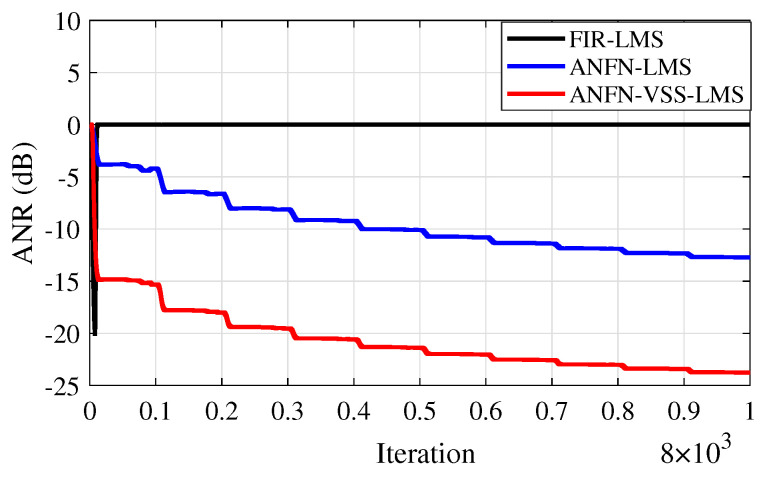
ANR curves of various methods.

**Case 4:** In this case, the noise source is modeled as the sum of a multi-sinusoidal signal with three frequency components: 20 Hz, 30 Hz, and 40 Hz. All other system parameters are set the same as in Case 3. The simulation results for this scenario are presented in Figure 20, Figure 21, Figure 22 and Figure 23. Figure 20a displays both the noise source and the noise after traversing the second-order nonlinear transfer function path in the time domain. This figure also shows that the noise amplitude in the control region varies significantly with respect to the noise source. Figure 20b presents the residual noise of the adaptive FIR-LMS model, indicating that the FIR-LMS method is ineffective when the system has nonlinearity and the signal amplitude changes abnormally, which leads to system instability. Figure 20c and Figure 20d present the residual noise of ANC systems using the ANFN-LMS and proposed ANFN-VSS-LMS methods, respectively. These figures show that the proposed ANFN-VSS-LMS controller outperforms the ANFN-LMS controller.

Figure 21 presents the simulation results in the frequency domain, where the blue solid line and the black dash line respectively represent the power spectrum of the noise source signal and the noise after passing through the nonlinear path. Here, it can be observed that numerous signals are generated at frequencies distinct from those of the noise source, which is the principal reason for the non-convergence of traditional ANC systems. The red dash line shows the spectrum power of the residual noise of the adaptive FIR-LMS filter, in which the amplitude of the high-frequency signals is quite large. The green solid line and the purple solid line respectively depict the residual signal spectrum of ANFN-LMS and ANFN-VSS-LMS controllers. This result verifies that the proposed ANFN control method is more reliable.

Figure 22a illustrates the learning weights of the ANFN-LMS controller, while Figure 22b shows those of the ANFN-VSS-LMS controller. These figures further demonstrate the reliability and effectiveness of employing a variable step size in the VSS-LMS algorithm.

Similar to cases 2 and 3, in this case, we also address the RMSE indices presented in Table 7 and the ANR shown in Figure 23 to further validate the reliability of the proposed system. Based on these results, we can confirm that the proposed ANC system demonstrates high reliability in nonlinear environments.

## 4. Conclusions

This paper presents a novel ANFN controller for nonlinear FF-ANC systems, which is designed to deal with nonlinearities in the primary path and noise sources. The proposed controller utilizes a fuzzy neural network, which combines the fuzzy logic system and the neural network to act as a nonlinear filter. Specifically, this paper also introduces a novel controller based on a variable step-size LMS algorithm to enhance the performance of the proposed system. The variable step-size parameters are dynamically updated online according to the residual error, taking a forgetting factor into account. A unified theoretical study is also proposed to analyze the stability of the ANC system and explore the convergence rate of the variable step-size parameters. Comparative simulation results are presented to verify the effectiveness of the proposed methods and illustrate the improved performance in terms of residual noise reduction. Based on the analysis of the system’s required computational load, reliable results from the system simulation have been obtained. In the near future, we will carry out a reliability assessment of the controller proposed in this study for highly nonlinear, real-world environments.

## Figures and Tables

**Table 1:** Computed by the FIR-LMS technique.

		Computation				
**Step**	**Computing Formula**	**(**·**)**	**(:)**	**(+)**	**(**−**)**	**exp(**·**)**
1	$y\left(n\right)=\sum_{k=0}^{N-1}w_{k}\left(n\right)x^{\prime }\left(n-k\right)$	*N*	—	N−1	—	—
2	$w\left(n+1\right)=w\left(n\right)+\mu e\left(n\right)x^{\prime }\left(n\right)$	N+1	—	*N*	—	—
Total		2N+1	—	2N−1	—	—

**Table 2:** Computed by the ANFN-LMS technique.

		Computation				
**Step**	**Computing Formula**	**(**·**)**	**(:)**	**(+)**	**(**−**)**	**exp(**·**)**
1	$y\left(n\right)=\sum_{i=0}^{N-1}w_{i}k_{i}\left(n\right)$	*N*	—	N−1	—	—
2	$k_{i}\left(n\right)=x\left(n-i\right)/O^{\left(3\right)}$	—	*N*	—	—	—
3	$O^{\left(3\right)}=\sum_{i=0}^{N-1}O_{i}^{\left(2\right)}$	—	—	*N*	—	—
4	$O_{i}^{\left(2\right)}=O_{i}^{\left(1\right)}\cdot ae^{\frac{-{\left(O_{i}^{\left(1\right)}-c\right)}^{2}}{2\sigma^{2}}}$	5N	*N*	—	*N*	*N*
5	$w\left(n+1\right)=w\left(n\right)+\mu e\left(n\right)\sum_{j=0}^{J}s_{j}k\left(n-j\right)$	N+1	—	*N*	—	—
Total		7N+1	2N	3N−1	*N*	*N*

**Table 3:** Computed by the ANFN-VSS-LMS technique.

		Computation				
**Step**	**Computing Formula**	**(**·**)**	**(:)**	**(+)**	**(**−**)**	**exp(**·**)**
1	$\mu^{\prime }\left(n+1\right)=\alpha \mu^{\prime }\left(n\right)+\gamma e^{2}\left(n\right)$	3	—	1	—	—
Total		7N+4	2N	3N	*N*	*N*

**Table 4:** Comparisons of computational costs.

Controllers	Multiplications	Divisions	Additions	Subtractions	Exponentiations
FxLMS	1*N* + 1	0	2*N* − 1	0	0
ANFN with FxLMS	7*N* + 1	2*N*	3*N* − 1	2*N*	*N*
ANFN with VSS-FxLMS	7*N* + 4	2*N*	3*N* − 1	2*N*	*N*

**Table 5:** Comparisons of the RMSE error index.

FIR-LMS	ANFN-LMS	ANFN-VSS-LMS
0.1968	0.1598	0.1045

**Table 6:** Comparisons of the RMSE error index.

FIR-LMS	ANFN-LMS	ANFN-VSS-LMS
6.937	0.3753	0.2617

**Table 7:** Comparisons of the RMSE error index.

FIR-LMS	ANFN-LMS	ANFN-VSS-LMS
116.8	0.6262	0.1858

## Data Availability

Data will be made available on request.

## Appendix A. The Proof of Theorem 1

**Proof.** First, consider the following Lyapunov function:

(A1) $$\begin{array}{c} V\left(n\right)=\frac{1}{2}e^{2}\left(n\right)=\frac{1}{2}{\left(d\left(n\right)-y^{\prime }\left(n\right)\right)}^{2}. \end{array}$$

Then the derivative of (A1) can be given by

(A2) $$\begin{array}{c} \begin{array}{cc} \Delta \;V(n) & =V(n+1)-V(n)\\ & =\frac{1}{2}\left(e^{2}\left(n+1\right)-e^{2}\left(n\right)\right)\\ & =\frac{1}{2}(e\left(n+1\right)-e\left(n\right)\left(e(n+1)+e(n)\right)\\ & =\frac{1}{2}\Delta e\left(n\right)\left(2e(n)+\Delta e(n)\right), \end{array} \end{array}$$

where Δe(n)=e(n+1)−e(n). Furthermore, it can be expressed as

(A3) $$\begin{array}{c} \begin{array}{cc} \Delta e(n) & ={\left(\frac{\partial e(n)}{\partial w(n)}\right)}^{T}\Delta w\left(n\right)\\ & ={\left(\frac{\partial e(n)}{\partial y^{\prime }\left(n\right)}\cdot \sum_{j=0}^{J}\left(\frac{\partial y^{\prime }\left(n\right)}{\partial y(n-j)}\cdot \frac{\partial y(n-j)}{\partial w(n)}\right)\right)}^{T}\cdot \left(w(n+1)-w(n)\right)\\ & =-\mu e\left(n\right){∥\sum_{j=0}^{J}s_{j}X\left(n-j\right)∥}^{2} \end{array}. \end{array}$$

By substituting (A3) into (A2), one has

(A4) $$\begin{array}{c} \begin{array}{cc} \Delta V(n) & =-\frac{1}{2}\;\mu e\left(n\right){∥\sum_{j=0}^{J}s_{j}X\left(n-j\right)∥}^{2}\cdot \left(2e\left(n\right)-\mu e\left(n\right){∥\sum_{j=0}^{J}s_{j}X\left(n-j\right)∥}^{2}\right)\\ & =-\frac{1}{2}\;\mu e^{2}\left(n\right){∥\sum_{j=0}^{J}s_{j}X\left(n-j\right)∥}^{2}\cdot \left(2-\mu {∥\sum_{j=0}^{J}s_{j}X\left(n-j\right)∥}^{2}\right) \end{array}. \end{array}$$

Consider the following inequality:

(A5) $$\begin{array}{c} 0<\mu \leq 2\;/∥\sum_{j=0}^{J}s_{j}X\left(n-j\right)∥. \end{array}$$

Then, it is clear that $V\left(n\right)=\frac{1}{2}e^{2}\left(n\right)\geq 0$. In addition, V(n)=0 if and only if e(n)=0. According to the Lyapunov theorem, the ANC system with the adaptive law (8) is stable under the condition (A5). □
